# ANGUSTIFOLIA, a Plant Homolog of CtBP/BARS Localizes to Stress Granules and Regulates Their Formation

**DOI:** 10.3389/fpls.2017.01004

**Published:** 2017-06-13

**Authors:** Hemal Bhasin, Martin Hülskamp

**Affiliations:** Botanik III, Biocenter, University of CologneCologne, Germany

**Keywords:** ANGUSTIFOLIA, leaf morphogenesis, trichomes, RNA, NADH

## Abstract

The *ANGUSTIFOLIA (AN)* gene in Arabidopsis is important for a plethora of morphological phenotypes. Recently, AN was also reported to be involved in responses to biotic and abiotic stresses. It encodes a homolog of the animal C-terminal binding proteins (CtBPs). In contrast to animal CtBPs, AN does not appear to function as a transcriptional co-repressor and instead functions outside nucleus where it might be involved in Golgi-associated membrane trafficking. In this study, we report a novel and unexplored role of AN as a component of stress granules (SGs). Interaction studies identified several RNA binding proteins that are associated with AN. AN co-localizes with several messenger ribonucleoprotein granule markers to SGs in a stress dependent manner. *an* mutants exhibit an altered SG formation. We provide evidence that the NAD(H) binding domain of AN is relevant in this context as proteins carrying mutations in this domain localize to a much higher degree to SGs and strongly reduce AN dimerization and its interaction with one interactor but not the others. Finally, we show that AN is a negative regulator of salt and osmotic stress responses in Arabidopsis suggesting a functional relevance in SGs.

## Introduction

Overall plant growth and development is based on the regulation of cell shape and cell division/expansion. Cell growth is tightly controlled by cell metabolism and thus by the availability of nutrients, cellular energy, and stress (Yuan et al., 2013). To understand the interplay between cell growth and metabolism, we are studying the *ANGUSTIFOLIA* (*AN*) gene in *Arabidopsis thaliana*. Mutations in *AN* lead to a plethora of growth phenotypes along with altered responses to stress and pathogen attack. Morphological phenotypes include narrow leaves, under-branched trichomes, spiral growth of roots, ovules, and siliques (Redei, 1962; Hulskamp et al., 1994; Tsukaya et al., 1994; Tsuge et al., 1996; Fulton et al., 2009; Bai et al., 2013). The narrow leaf phenotype is caused by a change in growth directionality of leaf cells and by a decreased number of cells in the width direction (Kim et al., 2002; Bai et al., 2010). The cell shape changes are correlated with changes in the microtubule cytoskeleton (Folkers et al., 2002; Kim et al., 2002; Sambade et al., 2014). More recently, it was reported that *an* mutants can cope better with drought stress and are more resistant to bacterial infection (Gachomo et al., 2013).

The *AN* gene encodes for a C-terminal-binding protein/brefeldin A-ADP ribosylated substrate (CtBP/BARS) homolog (Folkers et al., 2002; Kim et al., 2002). BARS are localized in the cytosol and have a role in membrane trafficking and Golgi fission (Spano et al., 1999; Colanzi et al., 2013). CtBP was first described as a human protein that interacts with C-terminal sequences Pro-X-Asp-Leu-Ser (PXDLS) of adenovirus E1A protein (Schaeper et al., 1995). One major function of CtBP family proteins is the transcriptional co-repression by binding transcriptional repressors (Chinnadurai, 2002, 2007). CtBP proteins belong to a family of NAD-dependent D2-hydroxy acid dehydrogenases (D2-HDHs) and contain a nicotinamide adenine dinucleotide (NAD) binding domain and a catalytic triad (His/Glu/Arg) that is conserved in all D2-HDHs. The NAD^+^ and NADH ratio in the cell controls the oligomerization of CtBP and its interaction with E1A. CtBPs have therefore been proposed to act as redox sensors (Zhang et al., 2002; Fjeld et al., 2003).

The molecular role of AN is still not clear. At its N-terminus, AN shares 32% homology with CtBPs while the C-terminus of AN contains a plant specific region. Like CtBPs, AN harbors a predicted NAD(H) binding domain in its N-terminus that, however, carries a few mutations at positions important for binding NAD(H). The overall structure of AN is conserved in plants and the functional conservation has been demonstrated for homologs from several species by the complementation of the *an* mutant phenotype (Cho et al., 2005; Lin et al., 2008; Minamisawa et al., 2011). A functional role of the NAD(H) binding domain is suggested by the observation that an amino acid exchange in the predicted NAD(H)-binding domain at position 170 from glycine to aspartic acid leads to a null-mutant phenotype (*an-doq*) (Fulton et al., 2009; Bai et al., 2013). A possible function for AN as a transcriptional co-repressor is supported by the finding that several genes are misregulated in *an* mutants (Kim et al., 2002; Fulton et al., 2009). On the other hand, a molecular function as a co-repressor appears to be unlikely as AN does not interact with adenovirus E1A protein and cannot repress the expression of reporter genes in transgenic drosophila embryos (Stern et al., 2007). In addition, it was shown that AN functions outside the nucleus and is possibly involved in membrane trafficking due to its partial *trans*-Golgi localization (Minamisawa et al., 2011).

AN fused to GFP under the control of the 35S or the native promoter localizes to a few very bright and big dots and a few smaller dots with much weaker florescence (Minamisawa et al., 2011). The smaller dots were shown to partially co-localize with the *trans-*Golgi network marker VHA-a1 (Minamisawa et al., 2011) and this localization was confirmed by immunoelectron microscopy using the α-AN antibody (Minamisawa et al., 2011). The bigger brighter dots were initially believed to be nuclei due to their large size (Folkers et al., 2002; Kim et al., 2002) but a subsequent report ruled them out as nuclei (Minamisawa et al., 2011). These dots were not affected by BFA treatment and did not localize with any of the tested organelle marker (Minamisawa et al., 2011). As these big aggregates were also not detected by immuno-electron microscopy using an α-AN antibody, they were proposed to be non-physiological aggregates (Minamisawa et al., 2011). For this reason, we did not consider the big bright AN aggregates in our present study.

The molecular function of AN largely remains elusive. One way to explore its function is the identification and analysis of its interaction partners. In this study, we report AN to interact with several stress granule components and to localize to stress granules (SGs). Cells respond to various stress conditions by rapid and global reprogramming of gene expression at the levels of transcription, post-transcription and translation. Post-transcription regulation is typically governed by RNA-binding proteins (RBPs) that are often associated with microscopically visible mRNA-ribonucleoprotein (mRNP) complexes (Bailey-Serres et al., 2009) including SGs and Processing bodies (P-bodies). RBPs dynamically interact with single/double strand RNAs and mediate mRNA maturation, localization, stability, decay and translation (Dreyfuss et al., 2002; Bailey-Serres et al., 2009; Ambrosone et al., 2012). SGs form in response to stress, and function as sequestering sites for untranslated mRNAs (Anderson and Kedersha, 2008, 2009; Buchan and Parker, 2009; Kedersha et al., 2013). They have been shown to be important for cell survival by preventing accumulation of misfolded proteins and sequestering some apoptosis regulatory factors (Arimoto et al., 2008; Kedersha et al., 2013; Arimoto-Matsuzaki et al., 2016). SGs are highly dynamic and undergo dissolution upon restoration to normal conditions. In mammals, SG assembly has been shown to be triggered by phosphorylation of eIF2a under stress conditions by stress sensing kinases which leads to a depletion the eIF2/ tRNAi Met/GTP ternary complex that is required for translation initiation (Kedersha et al., 2002; Kimball et al., 2003). RBPs such as the T-cell intracellular antigen 1 (TIA-1), Ras-GAP SH3 domain-binding protein (G3BP) and TRISTETRAPROLIN (TTP) promote SG formation through low affinity interactions between intrinsically disordered regions present in these proteins (Kedersha et al., 2013). In plants only few factors are known to control SG formation (Sorenson and Bailey-Serres, 2014; Gutierrez-Beltran et al., 2015). OLIGOURIDYLATE BINDING PROTEIN 1 (UBP1), RNA-BINDING PROTEIN 45 and 47 (RBP45/47), and PAB (poly-A binding) protein families were recognized as the plant RBPs most closely related to animal TIA-1 (Sorenson and Bailey-Serres, 2014). However, the mechanism of mRNP formation and their composition largely remains unexplored in plants. Several proteins with putative RNA binding or modification activity are unique to plants and hence might have a plant-specific function (Lorković and Barta, 2002; Lorkovic, 2009).

## Materials and Methods

### Plant Lines and Growth Conditions

Arabidopsis (*Arabidopsis thaliana*) plants were grown on soil at 24°C, 16 h light per day. The following lines were used: Landsberg *erecta* (Ler), Columbia-0 (Col-0), *an-2* (Kim et al., 2002), *an-1* (Kim et al., 2002), *an-doq* (Bai et al., 2013), 35S:PAB2-RFP [kindly provided by Prof. Julia Bailey-Serres, University of California, Riverside (Sorenson and Bailey-Serres, 2014)] and new lines established in this study including 35S:YFP-AN, 35S:AN-YFP, 35S:YFP-AN^DOQ1^, 35S:YFP-AN^GAD→V V A^, 35S:YFP-UBP1B, and 35S:YFP-EIF4E1. N-terminal YFP and C-terminal YFP fusions were created by recombination of the corresponding entry clones with the gateway destination vectors pENS-G-YFP and pEXS-G-YFP (Feys et al., 2005). Transformation of *Arabidopsis thaliana* was done by the Floral Dip method (Clough and Bent, 1998) and transgenic plants were selected in the T1 by Glufosinat (BASTA^®^).

### Constructs

CDSs of all the genes were amplified from Col-0 cDNA and cloned through BP recombination in pDONR201 or pDONR207 (Invitrogen), sequenced and introduced into destination vectors by LR reactions (Invitrogen). List of constructs and primers used in this study with the AGI code are provided in the Supplementary Table S3. Mutant entry clones for AN^DOQ^ and AN^GAD→V V A^ were created by site-directed mutagenesis. AN^DOQ^ has a G170→D mutation mimicking the *an-doq* allele (Bai et al., 2013). AN^GAD→V V A^ has mutations at three sites important for NADH binding: G170→V; A175→V and D→193A. For Lumier, the destination vectors pcDNA3-Rluc-GW and pTREXdest30 (Invitrogen) were used which enable the N-terminal fusion of *Renilla reniformis* and *Staphylococcus aureus* proteins, respectively, and were described in detail before (Pesch et al., 2013; Pesch et al., 2015). Protein fusions were expressed in the human HEK293TN cells.

### Transformation of Arabidopsis Cell Culture and Purification

Dark grown Arabidopsis cell culture (wild-type L*er*) was transfected with pENSG 35S:YFP-AN-strep transformed in *Agrobacterium* strain GV3101-pMP90RK as described before (Pesch et al., 2014) and grown at 22°C and 120 rpm in the dark for 5 days. The cells were harvested, pellets were crushed in liquid nitrogen, and protein crude extracts were prepared by homogenizing the cell pellets in 500 ml protein extraction buffer [50 mM Tris pH 7.5; 150 mM NaCl; 1 mM EDTA; 10% Glycerol; 5 mM DTT; 1% protease inhibitor cocktail (cOmpletes^TM^, Roche); phosphatase inhibitor cocktail (PhosSTOP^TM^, Roche); 1% Triton-X100]. After two centrifugation steps (12,000 rpm, 4°C, 20 min) the supernatant was incubated with Strep-Tactin^®^ MacroPrep (IBA) for 1 h followed by five washings (lysis buffer without Triton-X100 and Glycerol). The protein was eluted using 2.5 mM desthiobiotin containing elution buffer.

### Pulldown Experiment Using LUMIER

Luminescence Based Mammalian Interactome System (LUMIER) assays were done as described previously (Pesch et al., 2013, 2015). Luciferase activity was determined before (input) and after the pulldown. Cells transformed with empty vectors served as a negative control. Percentage pulldown efficiency was calculated by: [relative luminescence unit pulldown/(relative luminescence unit input)] × 100.

### Confocal Imaging

We used the Leica TCS-SP8/SPE confocal microscope equipped with the LCS software. Images were made using 20 or 40x water/oil immersion objectives. YFP was excited at 488 nm and detected at 510–560 nm. RFP was excited at 561 nm and detected in the 580–650 nm range. ImageJ was used for particle count/size analysis from maximum projections of Z series. Laser intensity, brightness and contrast settings were kept the same for an unbiased analysis for the particle count in wild-type and mutant backgrounds. For particle analysis, we considered granules between 0.185 and 5 μm^2^ to exclude the background and nuclear signal from the images. Co-localization analysis was performed by calculating Pearson’s coefficient (Adler and Parmryd, 2010; Dunn et al., 2011) using the JACOP plugin of ImageJ. Z-stack images were obtained and merged to maximum projection. For quality control, the threshold was set for each image manually such that background noise, but not distinct aggregates were eliminated. Analysis was performed on the entire image. Microscopic settings for the localization of AN were chosen to enable a safe visualization of the weakly labeled granules allowing signal saturation in the big, strongly fluorescent AN granules that were not considered in our analysis.

### Salt and Osmotic Stress Treatment of Seedlings

Seeds were sterilized and plated on 1/2 MS medium and stratified for 4 days at 4°C in the dark and transferred to normal growth conditions. After 5 days, seedlings were transferred to medium supplemented with 150/175 mM NaCl for analyzing salt tolerance and 200/250 mM mannitol for analyzing osmotic stress tolerance. Six and nine days after transfer to NaCl or mannitol media, seedlings were analyzed for tolerance to stress conditions by analyzing cotyledon greening and root length under stress/non-stress conditions. Root length was measured using ImageJ.

### Transient Expression Assay

Transfection of Arabidopsis leaves was performed by biolistic transformation (Mathur et al., 2003) and analyzed after 12–16 h by Confocal Laser Scanning Microscopy (CLSM).

### Yeast Two-Hybrid

pACT2 and pAS2 plasmids (Clontech) were used for the fusion of the proteins with GAL4 activation domain and GAL4 DNA binding domain respectively. Yeast two-hybrid assays were done as previously described (Gietz et al., 1995) and interactions were analyzed on synthetic dropout medium without leucine, tryptophan, and histidine supplemented with varying concentrations of 3-aminotriazole (3-AT).

### Mass Spectrometry

Mass spectrometry analysis was performed as described previously (Borgal et al., 2014; Hosseinibarkooie et al., 2016). Sample analysis was performed on an LTQ Orbitrap Discovery mass spectrometer (Thermo Scientific) coupled to an EASYnLC II nano-LC system (Proxeon, part of Thermo Scientific).

### Accession Numbers of Genes Expressed in Stable Lines

AN (AT1G01510), PAB2 (AT4G34110), UBP1B (AT1G17370), EIF4E1 (AT4G18040).

## Results

### AN Protein Physically Interacts with RNA Binding Proteins

In an attempt to understand the molecular function of AN in more detail, we searched for proteins interacting with AN using the yeast two-hybrid screens. Although we performed several screening rounds with the full length AN protein as a bait on different libraries, we isolated only two interactors, ANGUSTIFOLIA INTERACTING KINASE (AIK1, AT3g17750) and Asymmetric Leaf Enhancer 3 (AE3, AT5g05780). In parallel, we performed a pulldown experiment using an Arabidopsis cell culture expressing N-terminal YFP tagged AN with a Strep tag at the C-terminus. AN was affinity purified using Strep-tactin resin and co-purified proteins were analyzed by mass spectrometry. We identified 62 co-precipitated proteins from AN expressing cell cultures that were not found in the control cell culture (Supplementary Tables S1, S2). We noted that 12 co-purified proteins are predicted to be associated with RNA metabolism suggesting that AN might be involved in the regulation of the RNA metabolism (**Table 1**). To test this further, we studied the protein–protein interactions of AN with selected proteins in quantitative pulldown experiments in HEK cells (**Table 1**), using the LUMIER (Barrios-Rodiles et al., 2005; Pesch et al., 2013). In short, a Prot-A tagged protein is co-transfected with *Renilla reniformis* luciferase in HEK cells and the amount of the interacting protein is determined after co-immunoprecipitation by a quantitative analysis of the luminescence (Barrios-Rodiles et al., 2005). The AN protein was always used as a luciferase-fusion and the putative interactors as Prot-A fusions. We chose seven RNA associated proteins that we isolated in the AN pulldown and 16 well-established RBPs chosen from the literature for the interaction studies (**Table 1**). In addition, we included the yeast two-hybrid interactor AIK1 as it is homologous to dual-specificity tyrosine phosphorylation-regulated kinase 3 (DYRK3) which was shown to control the dissolution of SGs in mammals through its role in mTOR signaling (Wippich et al., 2013). As summarized in **Table 1**, we found direct interaction with several but not all RNA associated proteins including RNA BINDING PROTEIN 47B (RBP47B; AT3G19130), RNA BINDING PROTEIN 47C (RBP47C; AT1G47490), RNA BINDING PROTEIN 47C′ (RBP47C′; AT1G47500), EUKARYOTIC INITIATION FACTOR 4E1 (EIF4E1; AT4G18040), EIF(iso)4E (AT5G35620), TANDEM ZINC FINGER 3 (TZF3; AT4G29190), ARGONAUTE1 (AGO1; AT1G48410), POLY(A)BINDING PROTEIN 2 (PAB2; AT4G34110), ANGUSTIFOLIA INTERACTING KINASE (AIK1, AT3g17750).

**Table 1:** AN interacts with several RNA associated proteins.

Accession	Description	Reference	Interact in LUMIER	Mass spectrometry score	Fold change from background^∗^	Percentage pulldown
**Mass spectrometry**						
AT1G47500	POLYADENYLATE-BINDING PROTEIN RBP47C′	Weber et al., 2008; Gutierrez-Beltran et al., 2015; Lokdarshi et al., 2016	YES	17.57	3.07	0.29
AT1G29400	PROTEIN MEI2-LIKE 5 (ATML5)	Anderson and Hanson, 2005; Kaur et al., 2006	YES	33.62	5.04	0.47
AT4G13850	GLYCINE-RICH RNA-BINDING PROTEIN 2 (GRP2)	Mangeon et al., 2010	YES	7.23	2.52	0.24
AT2G38610	KH DOMAIN-CONTAINING PROTEIN	Lorković and Barta, 2002	YES	30.69	2.93	0.28
AT5G54900	POLYADENYLATE-BINDING PROTEIN RBP45A	Lorkovic et al., 2000	NO	13.83	1.04	0.15
AT3G20250	PUMILIO HOMOLOG 5 (PUM5)	Abbasi et al., 2014	NO	9.51	1.77	0.17
AT2G21660	GLYCINE-RICH RNA-BINDING PROTEIN 7 (GRP7)	Schmidt et al., 2010	NO	14.22	1.44	0.13
AT1G49600	POLYADENYLATE-BINDING PROTEIN RBP47A	Weber et al., 2008; Gutierrez-Beltran et al., 2015 Lokdarshi et al., 2016	N.D.	37.30	N.D.	N.D.
AT4G38680	COLD SHOCK PROTEIN 2 (CSP2)	Sasaki et al., 2007; Nakaminami et al., 2009; Ciuzan et al., 2015	N.D.	8.22	N.D.	N.D.
AT1G43190	POLYPYRIMIDINE TRACT-BINDING PROTEIN HOMOLOG 3 (PTBP3)	Stauffer et al., 2010; Ruhl et al., 2012	N.D.	11.10	N.D.	N.D.
AT3G20650	mRNA cap guanine-N7 methyltransferase 1	The Arabidopsis Information Resource (TAIR)	N.D.	11.19	N.D.	N.D.
AT1G56340	CALRETICULIN-1 (CRT1)	Carpio et al., 2010 (reports on SG localization/RNA association are based on mammalian studies)	N.D.	7.05	N.D.	N.D.
**Yeast two-hybrid**						
AT3G17750	PROTEIN KINASE SUPERFAMILY PROTEIN (AIK1)	The Arabidopsis Information Resource (TAIR)	YES	N.A.	223.30	35.97
**RBPs chosen from literature for interaction studies**						
AT3G19130	RNA BINDING PROTEIN 47B (RBP47B)	Weber et al., 2008; Gutierrez-Beltran et al., 2015; Lokdarshi et al., 2016	YES	N.A.	3.53	0.33
AT1G47490	RNA BINDING PROTEIN 47C (RBP47C)	Weber et al., 2008; Gutierrez-Beltran et al., 2015; Lokdarshi et al., 2016	YES	N.A.	4.97	0.46
AT4G34110	POLY(A) BINDING PROTEIN 2 (PAB2)	Sorenson and Bailey-Serres, 2014	YES	N.A.	7.67	0.71
AT4G18040	EUKARYOTIC INITIATION FACTOR 4E1 (EIF4E1)	Kawaguchi and Bailey-Serres, 2002; Weber et al., 2008; Gutierrez-Beltran et al., 2015	YES	N.A.	2.83	0.27
AT5G35620	EIF (ISO) 4E	Bailey-Serres et al., 2009; Hafrén et al., 2015	YES	N.A.	4.09	0.39
AT4G29190	TANDEM ZINC FINGER 3 (TZF3)	Lee et al., 2012	YES	N.A.	5.25	0.50
AT1G48410	ARGONAUTE1 (AGO1)	Carbonell et al., 2012; Arribas-Hernández et al., 2016	YES	N.A.	5.02	0.47
AT3G16640	TRANSLATIONALLY CONTROLLED TUMOR PROTEIN (TCTP)	Rinnerthaler et al., 2013 ^∗^localization to SGs based on yeast	YES	N.A.	3.12	0.28
AT1G66810	TANDEM CCCH ZINC FINGER (TZF) ATC3H14	Kim et al., 2014	YES	N.A.	4.92	0.47
AT1G54080	OLIGOURIDYLATE BINDING PROTEIN 1A (UBP1A)	Weber et al., 2008; Sorenson and Bailey-Serres, 2014	NO	N.A.	1.49	0.21
AT1G17370	OLIGOURIDYLATE BINDING PROTEIN 1B (UBP1B)	Weber et al., 2008; Sorenson and Bailey-Serres, 2014	NO	N.A.	0.86	0.08
AT3G14100	OLIGOURIDYLATE BINDING PROTEIN 1C (UBP1C)	Weber et al., 2008; Sorenson and Bailey-Serres, 2014	NO	N.A.	0.94	0.09
AT5G13570	DECAPPING PROTEIN 2 (DCP2)	Xu et al., 2006; Weber et al., 2008	NO	N.A.	1.63	0.13
AT1G08370	DECAPPING PROTEIN 1 (DCP1)	Xu et al., 2006; Weber et al., 2008	NO	N.A.	1.00	0.08
AT1G69440	ARGONAUTE7 (AGO7)	Carbonell et al., 2012	NO	N.A.	1.78	0.14
AT1G26110	DECAPPING PROTEIN 5 (DCP5)	Xu and Chua, 2009	NO	N.A.	1.09	0.08

*^∗^For the interaction analysis, only those proteins were considered as positive interactors which had higher than two times fold change from the background. N.D., not determined; N.A., not applicable.*

### AN Localizes to Cytosolic Granules upon Different Stress Treatments

**FIGURE 1:**
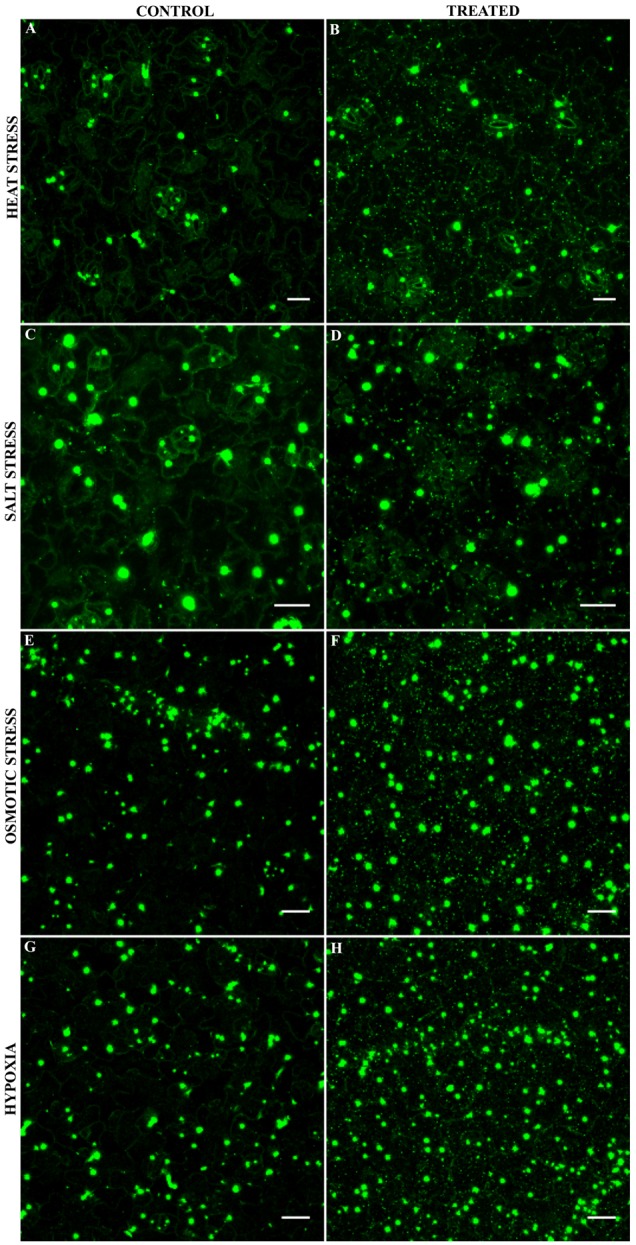
Localization of AN-YFP under different stress conditions. Images for comparison of AN localization before and after treatments were obtained from the same leaf. Microscopic settings were chosen to enable a good visualization of the weak dots, ignoring potential saturation of the big bright dots not considered here. Rosette leaf of a 3 week old plant before **(A)** and after 40 min heat stress at 39°C **(B)**. Cotyledons of 5–7 day old plants before **(C)** and 40 min after addition of 175 mM sodium chloride **(D)**. Cotyledons of 5–7 day old plants before **(E)** and 40 min after addition of 800 mM sorbitol **(F)**. Cotyledons of 5–7 day old plants before **(G)** and 40 min after addition 8 units/ml oxyrase for the induction of hypoxia **(H)**. Scale bars: 20 μm.

Our LUMIER data show that AN binds to several proteins localizing to mRNP complexes. mRNP complexes are typically formed under stress conditions (Kedersha et al., 2005; Weber et al., 2008). We therefore investigated the localization of AN before and after different stress treatments. Previous reports have demonstrated that 35S:AN-GFP shows the same intracellular localization as ANp:AN-GFP and completely rescues the *an* mutant phenotype (Minamisawa et al., 2011). In this study, we established two lines expressing either AN-YFP or YFP-AN under the 35S promoter in the *an-2* mutant background. These lines exhibit complete phenotypic rescue. Under normal conditions, both lines showed low cytoplasmic signal and one or few bright dots along with a few smaller dots showing weaker fluorescence, similar as reported before (Minamisawa et al., 2011). In a first experiment, we applied heat stress (39°C) to rosette leaves of AN-YFP plants for 40 min (Gutierrez-Beltran et al., 2015). When comparing leaves before and after the treatment we found the formation of many new cytosolic granules after heat stress (**Figures 1A,B**). The response to salt, osmotic and hypoxia was analyzed in the cotyledons of 5–7 days old seedlings grown on agar plates. Plants were transferred to depression slides and stress was induced by adding ½ MS solution containing 175 mM sodium chloride for salt stress conditions, 800 mM sorbitol (Wippich et al., 2013) for osmotic stress and 8 units/ml oxyrase for the induction of hypoxia (Sorenson and Bailey-Serres, 2014; Lokdarshi et al., 2016). All treatments caused the formation of many cytosolic granules (**Figures 1C–H**). Control experiments with ½ MS did not show a change in AN localization behavior (Supplementary Figure S1). We did not observe any differences in the recruitment behavior to granules between the C- and N- terminal fusions (AN-YFP and YFP-AN).

### AN Co-localizes with Several mRNP Granule Associated Proteins to Stress Granules upon Heat Stress

**FIGURE 2:**
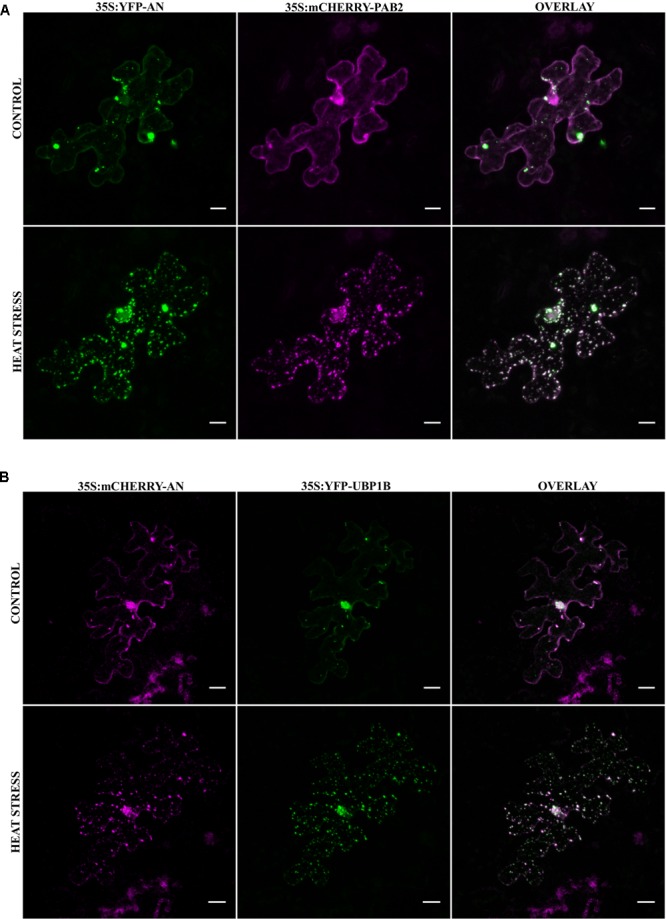
Co-localization of AN with RNA-binding proteins before and after heat stress. Co-localization studies were done in transiently transformed Arabidopsis rosette leaves by particle bombardment. **(A)** Single epidermal cell showing the localization of YFP-AN (green) and mCHERRY-PAB2 (red) before and after heat stress. Co-localization of the proteins appears white in the overlay. Scale bar: 10 μm. **(B)** Single epidermal cell showing the localization of mCHERRY-AN (red) and YFP-UBP1B (green) before and after heat stress. Co-localization of the proteins appears white in the overlay. Scale bar: 20 μm. Pearson coefficients of 0.73 ± 0.12 (*n* = 13) and 0.82 ± 0.07 (*n* = 14) were obtained for co-localization analysis of AN with PAB2 and UBP1B respectively.

The stress dependent formation of AN-labeled granules combined with our finding that AN protein binds to RNA-associated proteins prompted us to test, whether AN localizes to mRNP granules or SGs. Toward this end, we co-expressed established XFP-labeled SG markers with XFP-AN in single epidermal cells in Arabidopsis rosette leaves by particle bombardment. We selected four established SG markers: PAB2 (Sorenson and Bailey-Serres, 2014), UBP1B (Weber et al., 2008; Sorenson and Bailey-Serres, 2014), RBP47C (Weber et al., 2008; Lokdarshi et al., 2016) and EIF4E1 (Weber et al., 2008). For the analysis of the co-localization of PAB2 (Sorenson and Bailey-Serres, 2014) and UBP1B (Weber et al., 2008; Sorenson and Bailey-Serres, 2014) we analyzed the same cell before and after 40 min heat stress (39°C) (**Figure 2**). After heat stress, we found a prominent co-localization of AN with both RNP-markers in small granules (**Figures 2A,B**). Similar results were obtained for the co-localization of AN with RBP47C (Supplementary Figure S2A; Weber et al., 2008; Lokdarshi et al., 2016) and EIF4E1 (Supplementary Figure S2B; Weber et al., 2008). Together these data indicate that AN protein can be recruited to SGs in a heat stress dependent manner. To determine whether AN localizes specifically to SGs or more generally to mRNP granules, we studied its co-localization with P-body marker DCP1. While SGs are thought to function as sites for transient RNA storage, P-bodies serve as platforms for RNA processing and decay (Anderson and Kedersha, 2008). Toward this end, we co-expressed mCHERRY-AN, YFP-UBP1B and the P-body marker CFP-DCP1 (Weber et al., 2008) (Supplementary Figure S3). While AN co-localized well with UBP1B, we found no convincing co-localization with DCP1 (Supplementary Figure S3). Occasionally we noted an association of AN and DCP1 labeled granules similarly as observed for DCP1a and TIA-1 (Kedersha et al., 2005).

### Co-localization of AN with PAB2 in Transgenic Lines

**FIGURE 3:**
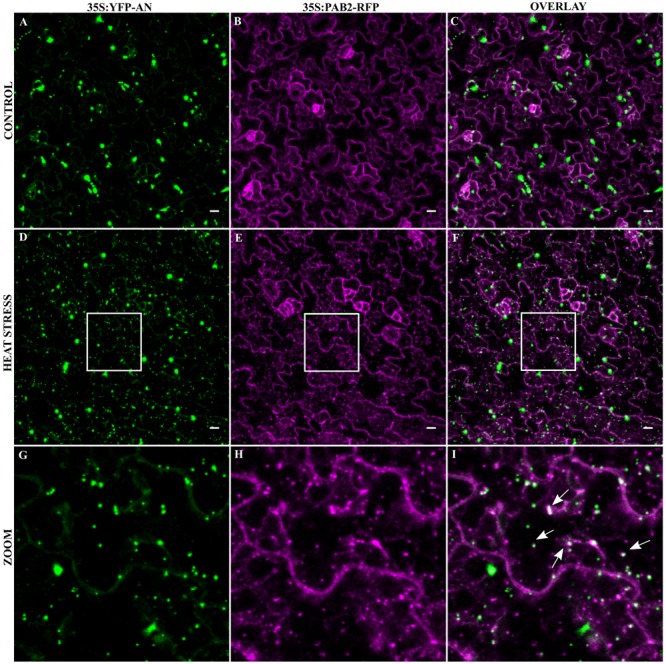
Co-localization of YFP-AN with PAB2-RFP in transgenic lines. Cotyledons from 5 to 7 days old seedlings of a 35S:YFP-AN and 35S:PAB2-RFP expressing plant were analyzed under normal conditions and after 40 min heat stress (39°C). The YFP and RFP channels and their overlay are shown. **(A–C)** Control leaf before stress. **(D–F)** Heat stressed leaf. **(G–I)** Higher magnification of the boxes indicated in **(D–F)**. Scale bar: 10 μm.

As the transformation of cells by particle bombardment may cause artificially high and thereby altered protein behavior, we confirmed the co-localization of the SG marker Poly-adenylate binding protein 2 (PAB2) with AN after stress in a stable transgenic line. PABP protein family is an integral SG component in mammals and is a universal marker for all SGs (Anderson and Kedersha, 2008). AtPAB2 has been shown to co-localize with another SG marker UBP1C (Sorenson and Bailey-Serres, 2014) and another member of PAB protein family, PAB8 has been used as a SG marker in Arabidopsis (Pomeranz et al., 2010). We crossed the YFP-AN line with the PAB2-RFP line (Sorenson and Bailey-Serres, 2014) and studied the protein co-localization in the F1 generation. As expected, YFP-AN was found in a big dot and few smaller dots and PAB2-RFP was cytoplasmic under normal conditions with (**Figures 3A–C**) and without YFP-AN (Supplementary Figure S4). We tested the response to heat stress in mature leaf cells and in the cotyledons of young seedlings. In both tissues we observed YFP-AN and/or PAB2-RFP positive granules (**Figures 3D–I**) after 40 min heat stress (39°C), which displayed a partial co-localization. The number of granules clearly showing both signals appeared to be lower than in the particle bombardment experiments (**Figures 3G–I**). This notion was confirmed by a lower Pearson coefficient of co-localization 0.30 ± 0.04 (*n* = 12 rosette leaves), 0.34 ± 0.4 (*n* = 8 cotyledons) in transgenic lines as compared to transient expression experiments (0.73 ± 0.12, *n* = 13). In our experiments we noted that both, the markers and AN can occur as single-labeled dots. Although we cannot resolve this at this stage, this can in principle result from different threshold levels for the detection of two proteins or be explained by qualitatively different granule populations. Taken together, these results show that heat stress dependent AN co-localization with SG markers occurs, although at different degrees, in transient expression assays as well as in transgenic lines.

### Density and Size of SGs Is Altered in *an* Mutants

**FIGURE 4:**
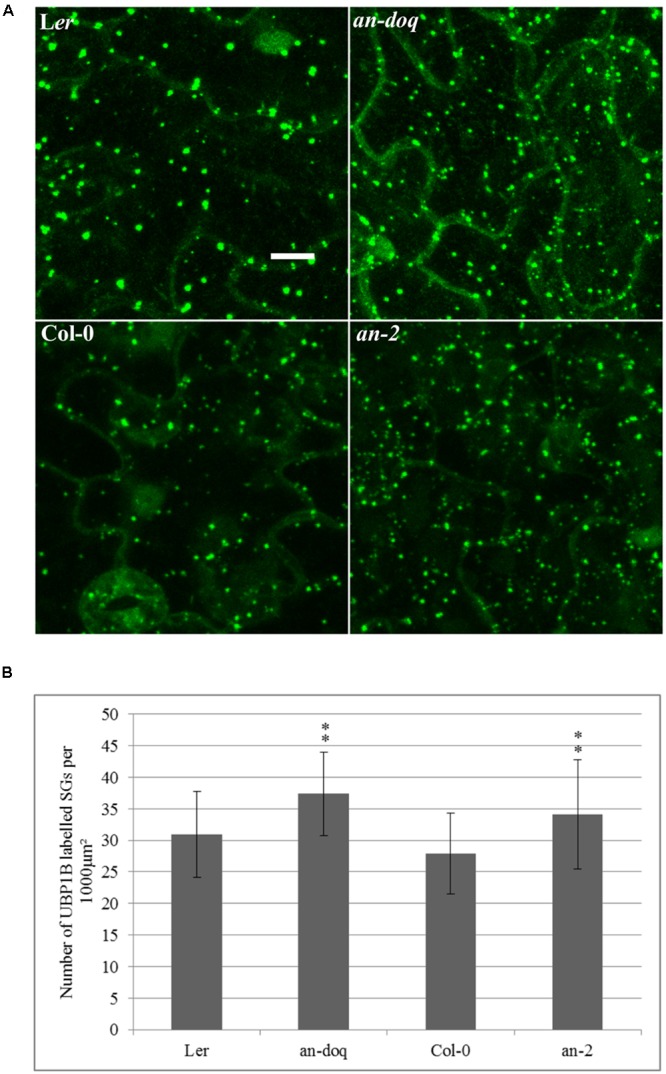
Stress granule (SG) size and number is altered in *an* mutants. Transgenic lines expressing 35S:YFP-UBP1B in L*er, an-doq*, Col-0 and *an-2* were analyzed after 40 min heat stress (39°C). The Z-stacked images were analyzed for the number and size of SGs using the ImageJ analyze particles tool. **(A)** Confocal microscopy images showing formation of UBP1B labeled granules in L*er, an-doq*, Col-0 and *an-2* after heat stress. Scale bar: 10 μm. **(B)** Number of UBP1B labeled SGs formed upon heat stress in wild-type and *an* mutant backgrounds. Graphs show mean number ± SD per 1000 μm^2^ area for *n* = 15∼30 seedlings; ^∗∗^*p* < 0.01; Student’s *t*-test.

In order to test, whether AN has a functional role in SG formation, we created transgenic marker lines expressing YFP-UBP1B and YFP-EIF4E1 under 35S promoter in *an-2, an*-*doq* and the respective wild-type backgrounds. In a first step, we quantified the number of YFP-UBP1B and YFP-EIF4E1 labeled SGs after 40 min heat stress (39°C). Cell sizes differ a lot in a given leaf and DNA content can range from 2C and 16C, it is therefore difficult to interpret the number of SGs per cell. For the comparison of wild-type and *an* mutants, we therefore studied the density per μm^2^. This should reflect the physiologically relevant cytoplasmic density of SGs in the cells. For both markers, we found a statistically significant higher number of SGs in both *an* alleles as compared to the wild-type (**Figure 4** and Supplementary Figure S5). This suggests that AN counteracts the formation of SGs. We additionally analyzed the size distribution of SGs in *an* mutants and wild-type. In both alleles we found a trend toward an increased number of smaller granules (Supplementary Figure S5). Thus, AN is required for the regulation of number and possibly the size of SGs.

### AN Proteins with Mutated NAD(H) Binding Domains Shows Higher Co-localization Levels with SGs

Our finding that the *an-doq* mutant, which has a point mutation in the NAD(H) binding domain, displays an altered number and size of SGs, prompted us to study the function of the NAD(H) binding domain in more detail. Toward this end we used two mutant proteins: AN^DOQ^, which is a mimic of *an-doq* mutant and AN^GAD→V V A^, which carries three amino acid exchanges in the NAD(H) domain potentially disrupting the NADH binding.

**FIGURE 5:**
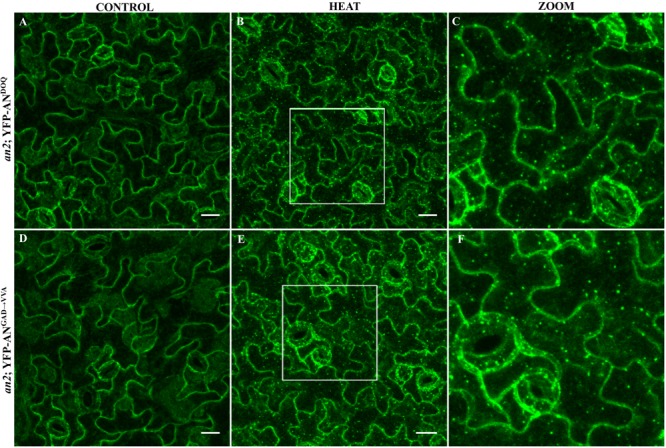
Localization of AN^DOQ^ and AN^GAD→V V A^ in the *an-2* mutant background. Transgenic lines expressing YFP-AN^DOQ^ and YFP-AN^GAD→V V A^ in the *an-2* mutant background. *an-2 YFP-AN^DOQ^* leaves without stress **(A)**, after 40 min heat stress (39°C) **(B)** and a higher magnification **(C)** of the box indicated in **(B)**. *an-2 YFP-AN^GAD→V V A^* leaves without stress **(D)**, after 40 min heat stress (39°C) **(E)** and a higher magnification **(F)** of the box indicated in **(E)**. Scale bar: 20 μm.

We expressed both mutant proteins as N-terminal YFP fusions in the *an-2* mutant under the control of the 35S promoter. Although *an-doq* mutant shows a null phenotype, overexpression of YFP-AN^DOQ^ rescued the *an-2* mutant morphological phenotypes suggesting that AN^DOQ^ protein has some residual activity. In contrast, YFP-AN^GAD→V V A^ overexpression showed weak rescue of the leaf length/width ratio and no rescue of the trichome phenotype (Supplementary Figure S6). Both proteins were cytoplasmic (**Figures 5A,D**). In contrast to wild-type AN protein (**Figure 1**), we found no dots under normal conditions. Upon heat stress, YFP-AN^DOQ^ and YFP-AN^GAD→V V A^ labeled granules were formed (**Figures 5B,C,E,F**). This suggests that the recruitment of both proteins to SGs can occur in the absence of wild-type AN protein.

**FIGURE 6:**
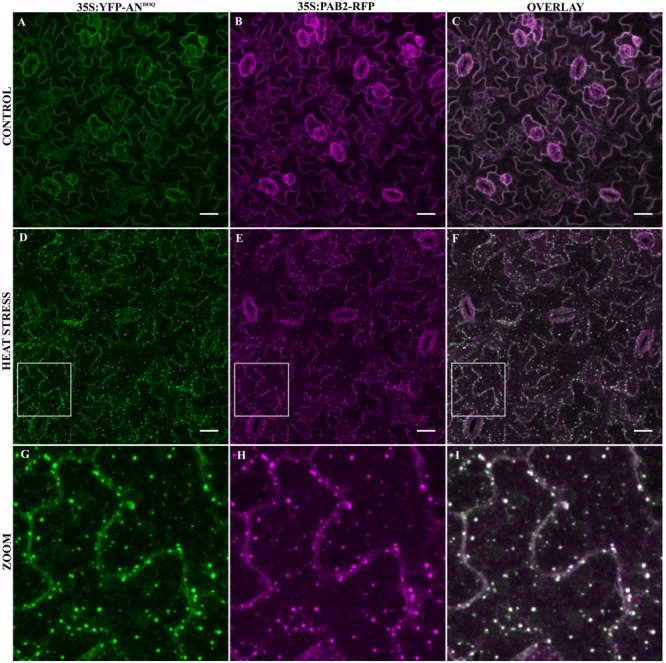
Co-localization of YFP-AN^DOQ^ with PAB2. Transgenic lines co-expressing YFP-AN^DOQ^ and PAB2-RFP. YFP-AN^DOQ^ and PAB2-RFP expressing leaf without stress **(A–C)**, after 40 min heat stress (39°C) **(D–F)** and a higher magnification **(G–I)** of the box indicated in **(D–F)**. Scale bar: 20 μm.

In a next step, we analyzed the co-localization of YFP-AN^DOQ^ and YFP-AN^GAD→V V A^ with the SG marker PAB2 in double transgenic lines. Without stress, we observed only cytoplasmic signals (**Figures 6A–C** and Supplementary Figures S7A–C). After heat stress, PAB2-RFP and YFP-AN^DOQ^ and YFP-AN^GAD→V V A^ positive granules were found (**Figures 6D–I** and Supplementary Figures S7D–I). Strikingly, YFP-AN^DOQ^ and YFP-AN^GAD→V V A^ signals appeared to almost completely co-localize with PAB2-RFP in contrast to the partial co-localization observed in case of wild-type protein (**Figure 3**). This visual impression was confirmed by a much higher Pearson coefficients of YFP-AN^DOQ^ (0.75 ± 0.06) and YFP-AN^GAD→V V A^ (0.77 ± 0.09) with PAB2-RFP as compared to wild-type AN (Pearson coefficient: 0.30 ± 0.04).

### The NAD(H) Domain Is Important for Differential Protein Interactions of AN Protein

**FIGURE 7:**
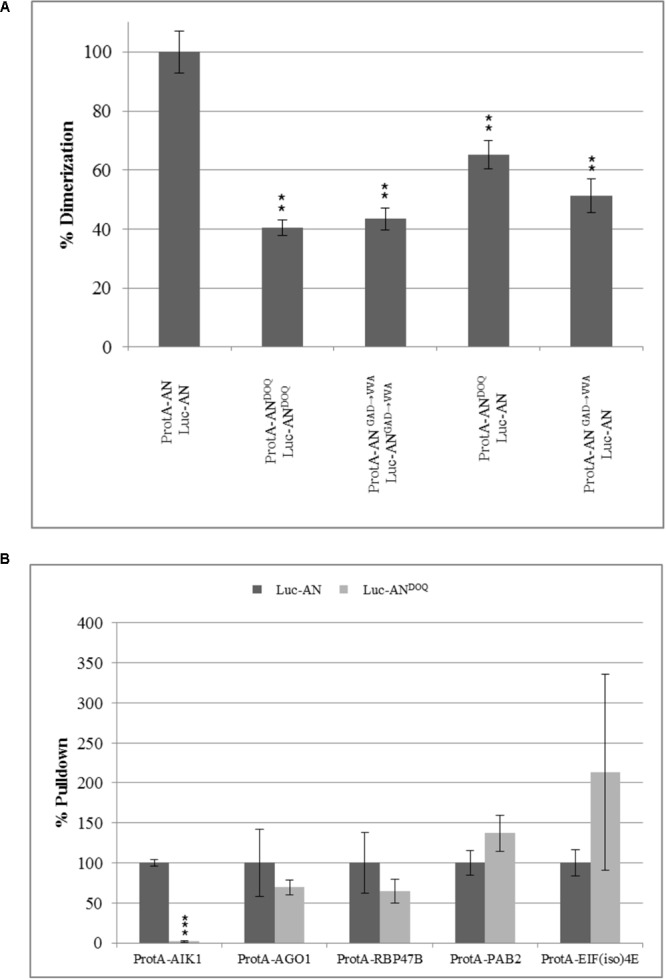
Point mutations in NADH binding domain lead to compromised dimerization rates and a selective reduction in interaction with AIK1. Pulldown values were plotted as percentage relative to the input and normalized to wild-type pulldown rates which was set as 100%. **(A)** Percentage dimerization efficiency of AN^DOQ^ and AN^GAD→V V A^ proteins as compared to wild-type AN. Dimerization is significantly reduced for AN^DOQ^ and AN^GAD→V V A^. Data is relative to wild-type which is set as 100%. Graph summarizes different experiments and standard deviation seen for the wild-type is highest seen in the experiments. Data are mean ± SD (*n* = 3). **(B)** Interaction efficiency of AN and AN^DOQ^ with some selected proteins. Interaction of AN^DOQ^ with AIK1 is significantly reduced whereas we found no significant differences to wild-type AN for the interaction with AGO1, RBP47B, PAB2 and EIF(iso)4E. ^∗∗^*p* < 0.01; Student’s *t*-test.

Previous reports on the role of the NAD(H) domain of CtBP in regulating its protein–protein interactions (Balasubramanian et al., 2003; Fjeld et al., 2003) prompted us to study the dimerization and interaction ability of AN^DOQ^ and AN^GAD→V V A^ proteins. In yeast two-hybrid assays, the dimerization of wild-type AN was observed at 3AT concentrations up to 30 mM whereas we detected no dimerization between AN^DOQ^ proteins (Supplementary Figure S8). To independently confirm this result, we used the LUMIER assay. Here we found a marked reduction of the interaction between AN^DOQ^ proteins (**Figure 7A**). Similar results were obtained for the AN^GAD→V V A^ protein (**Figure 7A**). In order to determine, whether the NAD(H) domain mutant proteins are generally impaired in protein–protein interactions or whether only specific protein interactions depend on this domain, we studied the interaction of AN^DOQ^ with five AN-interactors including AIK1, AGO1, RBP47B, PAB2, and EIF(iso)4E. We found a drastically reduced interaction of AN^DOQ^ with AIK1 (**Figure 7B**) (reduced to 1.6% of wild-type AN). However, we did not detect significant changes in interaction ability with AGO1, RBP47B, PAB2 and EIF(iso)4E (**Figure 7B**). This indicates that the NAD(H) binding domain of AN is relevant for some but not all interactions.

### AN Is Involved in Salt and Osmotic Stress Responses in Plants

**FIGURE 8:**
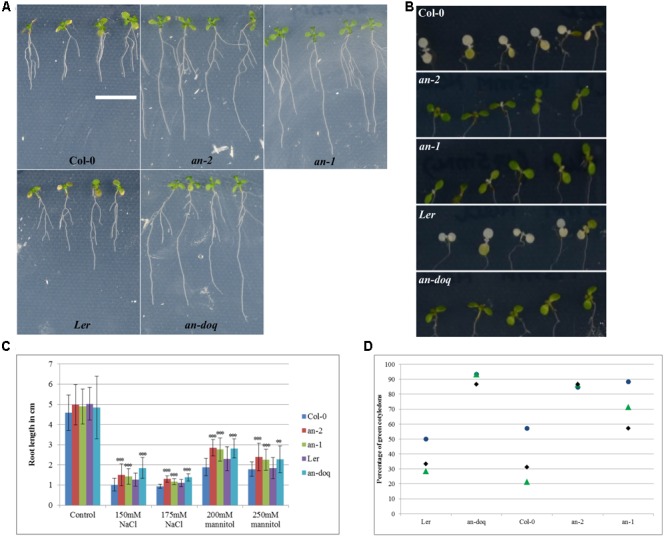
*an* mutants are hyposensitive to salt and osmotic stress. 5 day old plants grown on ½ MS were transferred to ½ MS containing different concentrations of NaCl and mannitol. Responses were assessed on the basis of primary root length growth and cotyledon greening. **(A)** Seedlings 9 days after the transfer to 250 mM mannitol containing media, Scale bar: 1 cm. **(B)** Cotyledons of seedlings 6 days after transfer to 175 mM NaCl containing media. **(C)** Root length of seedlings 9 days after the transfer to NaCl/Mannitol containing media. More than 40 seedlings were analyzed for each condition. ^∗∗^*p* < 0.01, ^∗∗∗^*p* < 0.001, Student’s *t*-test. **(D)** Dot plot distribution showing cotyledon greening percentage for seedlings grown on ½ MS + 175 mM NaCl after 6 days. Each data point represents 13–15 seedlings per condition. Triplicates are presented for each growth condition.

Our findings that AN is recruited to SGs upon stress treatment and its role in regulating SG number and size raised the question whether AN has, in addition to the well-reported cell morphogenesis phenotypes, also a role in stress responses. First evidence for a role of AN in biotic and abiotic stress tolerance was reported by Gachomo et al. (2013). In this study, we chose salt and osmotic stress: two conditions under which we observed AN recruitment to SGs and for which standardized treatments are well-established. *an* mutants and wild-type plants were grown on ½ MS plate supplemented with different concentration of salt or mannitol. After 6–9 days of growth, the primary root length and the greening of the cotyledons was compared between non-treated and stress treated seedlings. At 150 and 175 mM NaCl concentrations *an* mutants displayed hyposensitivity to salt stress (**Figure 8**). *an* mutants showed longer roots and greener cotyledons as compared to wild-type. Similarly, *an* mutants were less sensitive than wild-type to 200 and 250 mM mannitol treatments (**Figure 8**).

## Discussion

In this study, we provide several lines of evidence suggesting that AN has a role as a component of SGs. First, AN is associated with several RBPs *in vivo* and directly interacts with some of them (**Table 1** and Supplementary Table S1). Second, AN co-localizes with RBPs to SGs upon stress (**Figures 2, 3** and Supplementary Figure S2). Third, AN regulates the formation of SGs (**Figure 4** and Supplementary Figure S5). The novel interaction and localization behavior of AN hence suggests that it acts as a post-transcriptional regulator.

### Possible Role of AN in Post-transcriptional Regulation

How can these novel findings be integrated to the current concepts on the molecular function of AN? Function and localization of AN has been controversial since its discovery. Some reports suggest it to be involved in transcriptional control similar as known for its mammalian homolog CtBP (Chinnadurai, 2007). In support of this, microarray analysis revealed differential gene expression between wild-type and *an* mutants in Arabidopsis (Kim et al., 2002; Fulton et al., 2009; Bai et al., 2013). This view is challenged by the findings that AN cannot rescue drosophila CtBP mutants (Stern et al., 2007) and functions outside the nucleus where it partially localizes to the *trans-*Golgi network (Minamisawa et al., 2011). Consistent with this, we found various membrane trafficking proteins in our pulldown experiments that are associated with AN (Supplementary Table S1) such as VPS34 and KEULE. Thus, AN appears to be associated with both membrane trafficking components as well as with mRNP components suggesting a dual function in the cytoplasm.

A possible role of AN in SGs is suggested by its interaction with various SG components and its localization to SGs after various stress treatments. The interactions of AN to SG components is also seen under non-stress conditions in our assays. This suggests that AN might generally interact with SG components under non-stress and stress conditions also in plant cells. Given that various SG proteins are involved in translational processes, it is possible that AN functions in the translational machinery. This would provide an elegant explanation for its broad range of functions in development, morphogenesis and biotic/abiotic stress responses. Nevertheless, our finding that SG formation is altered in *an* mutants indicates that AN has a relevant function in SGs (**Figure 4** and Supplementary Figure S5).

### Function of the NAD(H) Binding Domain of AN

A major mechanism by which CtBPs regulate transcriptional events is by their ability to bind NAD(H) which results in a conformational change in CtBPs, in turn effecting its binding to interaction partners (Balasubramanian et al., 2003; Kuppuswamy et al., 2008; Nardini et al., 2009; Zhang and Arnosti, 2011). CtBPs are proposed to act as redox sensors (Zhang et al., 2002; Fjeld et al., 2003) due to their reported higher affinity for reduced NADH dinucleotide over NAD+, hence linking cellular redox state to the transcriptional output. Also, the disruption of the NAD(H) binding site in CtBPs lead to reduced or abolished dimerization rates (Nardini et al., 2009; Madison et al., 2013) and dimerization in turn is important for the regulation of co-transcriptional activity of CtBP (Madison et al., 2013). A functional role of the AN NADH binding domain is suggested by the finding that a point mutation in the NAD(H) domain results in a complete lack of function phenotype in *an-doq* allele (Bai et al., 2013). In this study, we found the same cellular phenotype for the stress dependent formation of SGs in *an-2* and *an-doq* allele (**Figure 4** and Supplementary Figure S5) and observed hyposensitivity of *an-2* and *an-doq* alleles to salt and osmotic stress (**Figure 8**). This suggests that the NAD(H) binding domain is also important for AN function in stress responses.

As experimental evidence for a binding of NAD(H) to AN is lacking, we cannot conclude whether the AN^DOQ^/AN^rmGAD→V V A^ mutations lead to the predicted reduced or no binding of NAD(H) to AN or whether they disturb the functions of the domain otherwise (e.g., stability, conformation, interaction ability). As the point mutations specifically target the positions predicted to be relevant for NAD(H) binding, it is reasonable to assume that AN activity is regulated by binding to NAD(H). As *an-doq* mutants display a null phenotype we propose that the NAD(H) bound form is the active form. The findings that overexpression of the AN^DOQ^ protein can fully rescue the *an* mutant trichome and leaf phenotypes and that overexpression of the AN^GAD→V V A^ protein can partially rescue the leaf length/width phenotype (Supplementary Figure S6) can be explained by some residual activity of the mutant proteins and that this reduced activity can be compensated by providing excess protein levels.

### Possible Role of AN in SGs

Our data suggest that the recruitment of AN to SGs does not depend on the NAD(H) domain as YFP-AN^DOQ^ and YFP-AN^GAD→V V A^ mutant proteins localize to SGs in the absence of wild-type protein (**Figure 5**). Both mutant proteins are evenly distributed in the cytoplasm (**Figure 5**). Big dots as observed with wild-type AN were not found, possibly because they represent non-physiological aggregates that do not form because the dimerization is strongly reduced in the mutant AN proteins (**Figure 7A**). The increased number of SGs in *an* mutants suggests that binding of the active form of AN counteracts the formation of SGs. It is conceivable that binding of the active form of AN is very transient and that this population of AN molecules cannot be seen in our co-localization experiments. As a consequence we would see only the inactive form of AN at SGs. These considerations would explain that AN^DOQ^ and AN^GAD→V V A^ show close to 100% co-localization with PAB2 labeled SGs while wild-type AN exhibits partial co-localization (**Figure 6** and Supplementary Figure S7). Along the same line, this scenario can explain the higher co-localization levels of wild-type AN with PAB2 in bombardment experiments as compared to transgenic lines as the high levels of AN reached in bombardment experiments may result in a larger population of inactive AN molecules, due to the limited availability of cellular NADH pools. What could be the role of AN in SGs? The moderately increased number of SGs in *an* mutants indicates that AN can counteract their formation not as a key regulator but rather as a modulator. One attractive possibility is that AN could fine tune SG formation in a redox dependent manner by its ability to monitor the NAD^+^/NADH ratio similar as shown for CtBPs.

### Regulation of AN Binding to Other Proteins by the NAD(H) Domain

A molecular basis for the functionality of NAD(H) binding domain is offered by the differential interaction of AN^DOQ^ with itself and other proteins. While some interactions are not effected by point mutations in the NAD(H) domain, the dimerization and its interaction with AIK1 is severely reduced (**Figure 7**). This suggests that the NADH binding domain mediates specific binding of AN to target proteins, thereby regulating their function in RBPs. Biotic and abiotic stresses can lead to an increase in reactive oxygen species (ROS) levels within a cell, leading to redox imbalance and oxidative stress (Hossain et al., 2015) that in turn could be sensed by the NADH binding domain of AN. We therefore speculate that AN function is governed by the redox status of the cell which in turn controls its interaction with specific SG associated proteins and thereby, the function of SGs. This is analogous to the function of Enhancer of decapping 3 (EDC3) and Apoptosis inducing factor (AIF) in mammals, which has been postulated to control mRNP granule formation in a redox dependent manner (Candé et al., 2004; Walters et al., 2014). A structural feature known to mediate aggregation of proteins into RNA granules is the presence of an intrinsically disordered/low complexity region (Kedersha et al., 2013). Using IUPred^1^ we found an intrinsically disordered region in AN between amino acid 342 and 487 at the N-terminus. It is therefore possible that AN can associate with SGs through such intrinsically disordered regions.

### Role of AN in SGs and Stress Responses in *an* Mutants

Is the association of AN with mRNP granules and its role in mRNP granule formation functionally significant? AN is involved in different stress responses as *an* mutants are more resistant than wild-type to dehydration stress and pathogen attack (Gachomo et al., 2013). More specifically, we observed a hyposensitivity of *an* mutants as compared to wild-type for two stress treatments triggering a re-localization of AN- salt and osmotic stress (**Figure 8**). These data suggest that a role of AN in stress responses can be explained by a function as a component of SGs, which are typically formed in response to stress. Examples for a correlation between SG formation and stress response phenotype include the SG components UBP1B and Tudor staphylococcal nuclease (TSN). UBP1B has been shown to be required for SG formation in plants (Weber et al., 2008) and *ubp1b* mutants are hypersensitive to salt and osmotic stress responses (McCue et al., 2012). Similarly, *tsn1tsn2* mutants show reduced number of SGs and are hypersensitive to heat stress (Gutierrez-Beltran et al., 2015). The novel findings hence suggest a role of AN as a post-transcriptional regulator, rather than a transcriptional repressor. An attractive scenario would be a role of AN in balancing growth and metabolism by sensing the cellular redox states.

## Conclusion

Our findings suggest that AN is involved in the coordination of cellular stress responses such that it regulates SG formation and possibly dissolution in a redox dependent manner. An interesting perspective for the future will be to identify direct molecular targets of AN, through which it regulates SG formation. It will also be interesting to test whether a function of AN in SGs is evolutionary conserved in CtBPs given that CtBPs are potential therapeutic targets for cancer treatment and that some SG signaling proteins have been associated with tumor progression (Somasekharan et al., 2015).

## Author Contributions

HB designed, planned and performed the experiments, analyzed the data and wrote the manuscript. MH supervised the project and wrote the manuscript.

## Conflict of Interest Statement

The authors declare that the research was conducted in the absence of any commercial or financial relationships that could be construed as a potential conflict of interest. The reviewer TH and handling Editor declared their shared affiliation, and the handling Editor states that the process met the standards of a fair and objective review.

## Footnotes

1 http://iupred.enzim.hu/
