# Supplementary material for: ANGUSTIFOLIA, a Plant Homolog of CtBP/BARS Localizes to Stress Granules and Regulates Their Formation
Source: Front Plant Sci. 2017 Jun 13;8:1004. doi: 10.3389/fpls.2017.01004 (PMC5469197; doi:10.3389/fpls.2017.01004)
Supplement: Supplementary file 4 [file Image_1.pdf]

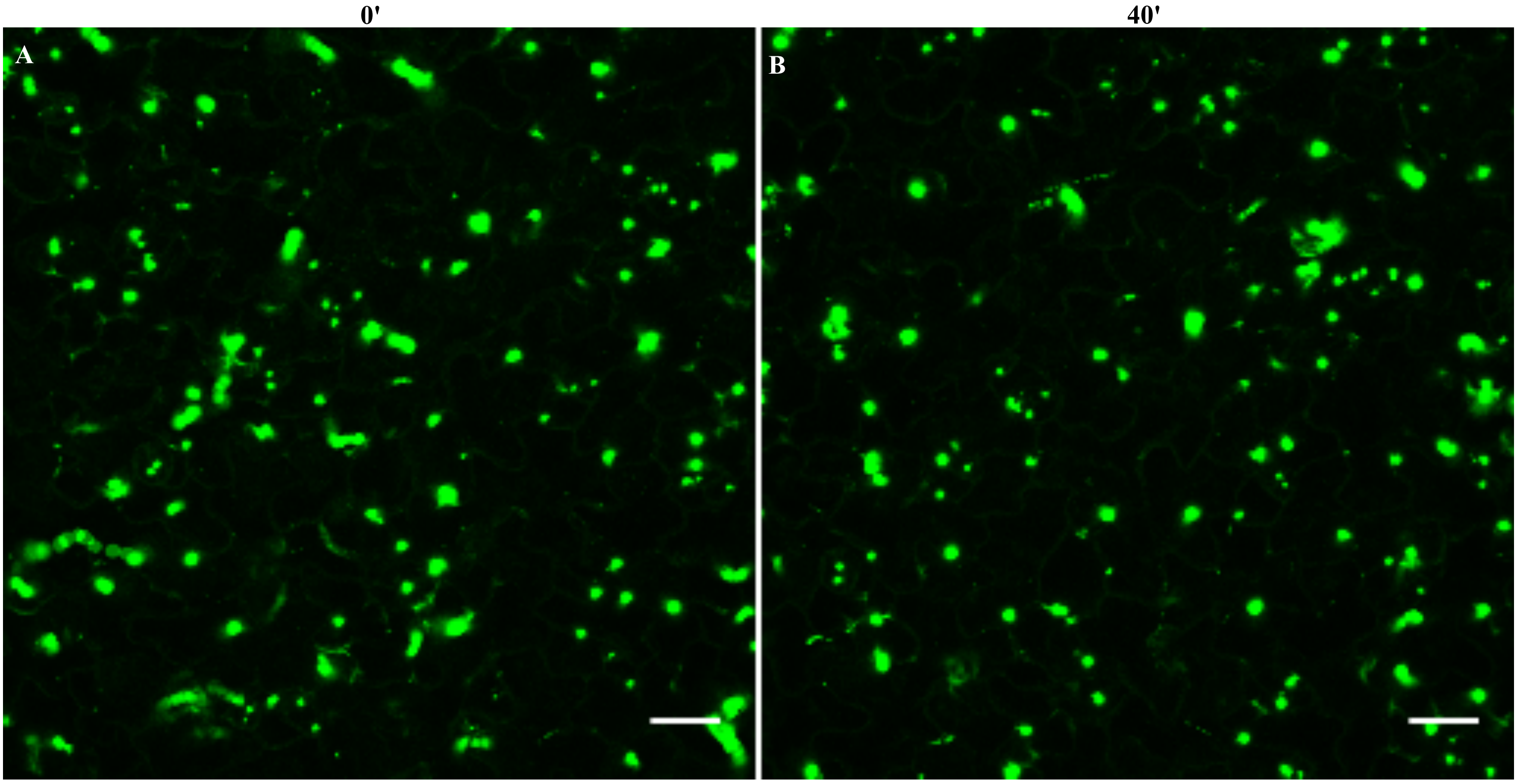

Figure S1: Localization of AN-YFP without stress

Images for comparison of AN localization before and after treatments were obtained from the same leaf. A,B) Cotyledons of five to seven day old plants treated with ½ MS (as a control) immediately after addition of the solution (A) and after 40 minutes (B). Scale bars: 20µm.
