# Supplementary material for: ANGUSTIFOLIA, a Plant Homolog of CtBP/BARS Localizes to Stress Granules and Regulates Their Formation
Source: Front Plant Sci. 2017 Jun 13;8:1004. doi: 10.3389/fpls.2017.01004 (PMC5469197; doi:10.3389/fpls.2017.01004)
Supplement: Supplementary file 5 [file Image_2.pdf]

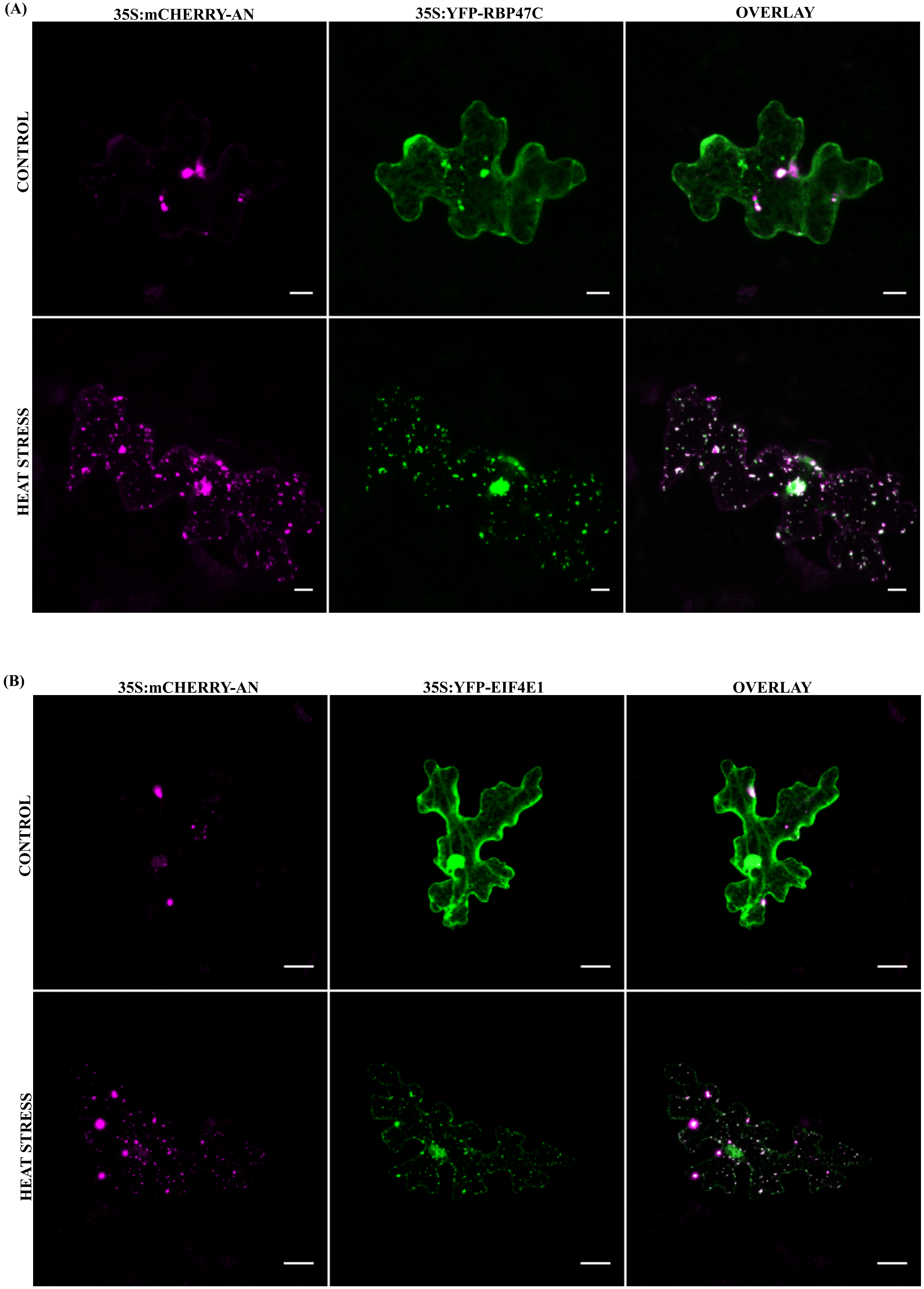
(A)
35S:mCHERRY-AN
35S:YFP-RBP47C
OVERLAY
CONTROL
HEAT STRESS
(B)
35S:mCHERRY-AN
35S:YFP-EIF4E1
OVERLAY
CONTROL
HEAT STRESS

Figure S2: Co-localization of AN with RNA-binding proteins with and without heat stress.

Co-localization studies were done in transiently transformed Arabidopsis rosette leaves by particle bombardment. A) mCHERRY-AN (red) and YFP-RBP47C (green) before and after heat stress. Co-localization of the proteins appears white in the overlay. Scale bar: 10 µm. B) mCHERRY-AN (red) and YFP- EIF4E1 (green) before and after heat stress. Co-localization of the proteins appears white in the overlay. Pearson coefficients of 0.64±0.11 (n=14) and 0.62±0.07 (n=8) were obtained for RBP47C and EIF4E1 respectively. Scale bar: 20 µm.
