# Supplementary material for: ANGUSTIFOLIA, a Plant Homolog of CtBP/BARS Localizes to Stress Granules and Regulates Their Formation
Source: Front Plant Sci. 2017 Jun 13;8:1004. doi: 10.3389/fpls.2017.01004 (PMC5469197; doi:10.3389/fpls.2017.01004)
Supplement: Supplementary file 6 [file Image_3.pdf]

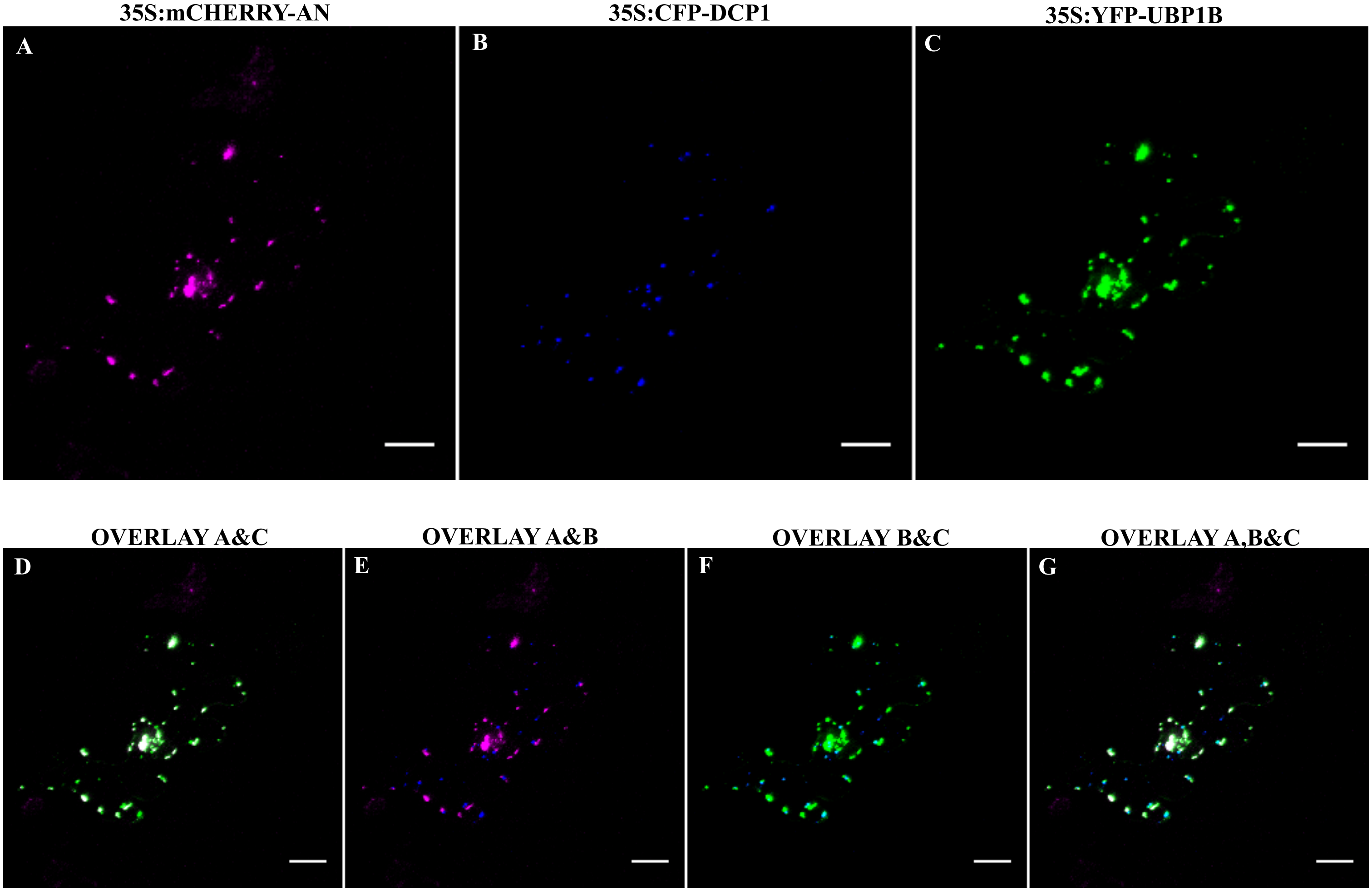

Figure S3: Co-localization of AN with UBP1 and DCP1

Co-localization was studied in transiently transformed Arabidopsis rosette leaves after 40 minute heat stress (39°C). A) mCHERRY-AN (magenta). B) CFP-DCP1 (blue) C) YFP-UBP1 (green) D) Overlay mCHERRY-AN and YFP-UBP1. E) Overlay mCHERRY-AN CFP-DCP1. F) Overlay YFP-UBP1 and CFP-DCP1. G) Overlay mCHERRY-AN, YFP-UBP1 and CFP-DCP1. Scale bar: 20 µm.
