# Supplementary material for: ANGUSTIFOLIA, a Plant Homolog of CtBP/BARS Localizes to Stress Granules and Regulates Their Formation
Source: Front Plant Sci. 2017 Jun 13;8:1004. doi: 10.3389/fpls.2017.01004 (PMC5469197; doi:10.3389/fpls.2017.01004)
Supplement: Supplementary file 7 [file Image_4.pdf]

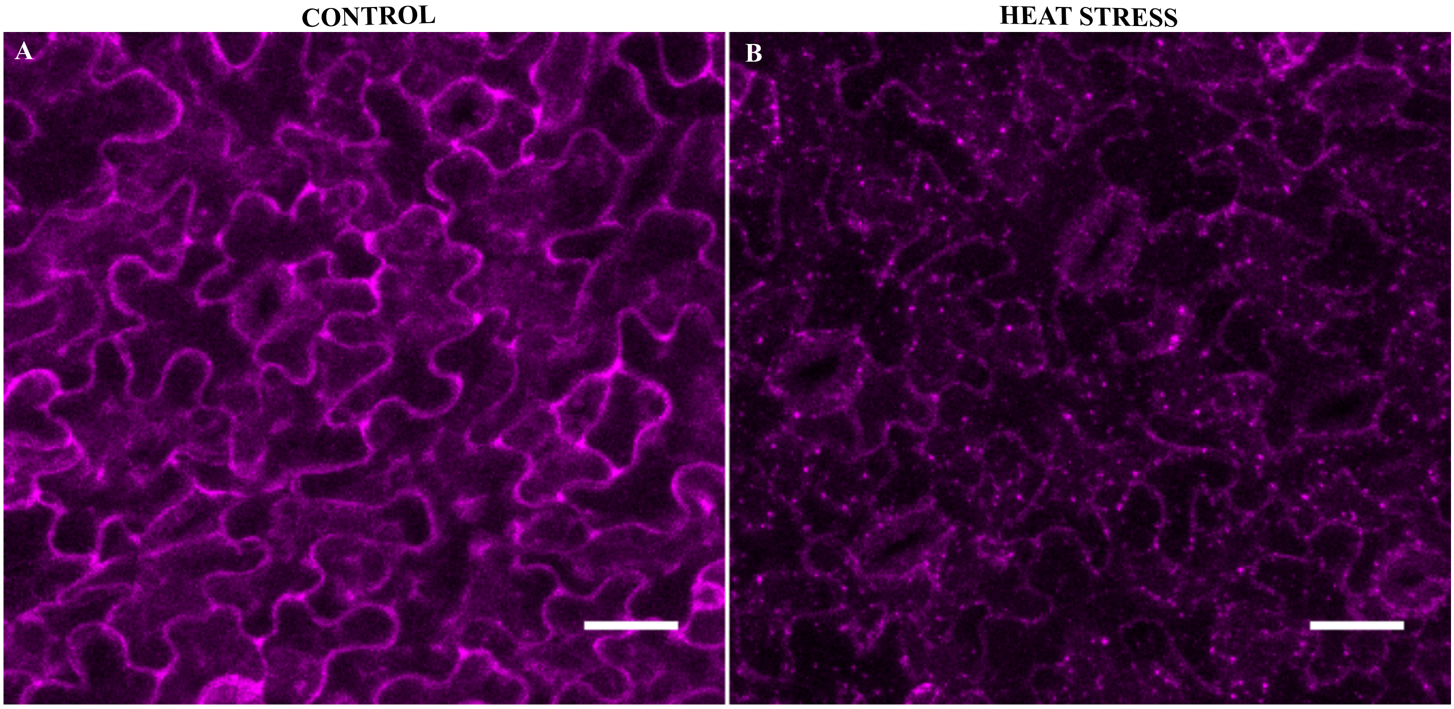

Figure S4: Localization of PAB2-RFP in transgenic lines
Cotyledons from 5-7 days old seedlings of a 35S:PAB2-RFP plants were analyzed under normal conditions and after 40 min heat stress (39°C). A) Control leaf without stress. B) Heat stress sample. Scale bar: 20µm.
