# Supplementary material for: ANGUSTIFOLIA, a Plant Homolog of CtBP/BARS Localizes to Stress Granules and Regulates Their Formation
Source: Front Plant Sci. 2017 Jun 13;8:1004. doi: 10.3389/fpls.2017.01004 (PMC5469197; doi:10.3389/fpls.2017.01004)
Supplement: Supplementary file 8 [file Image_5.pdf]

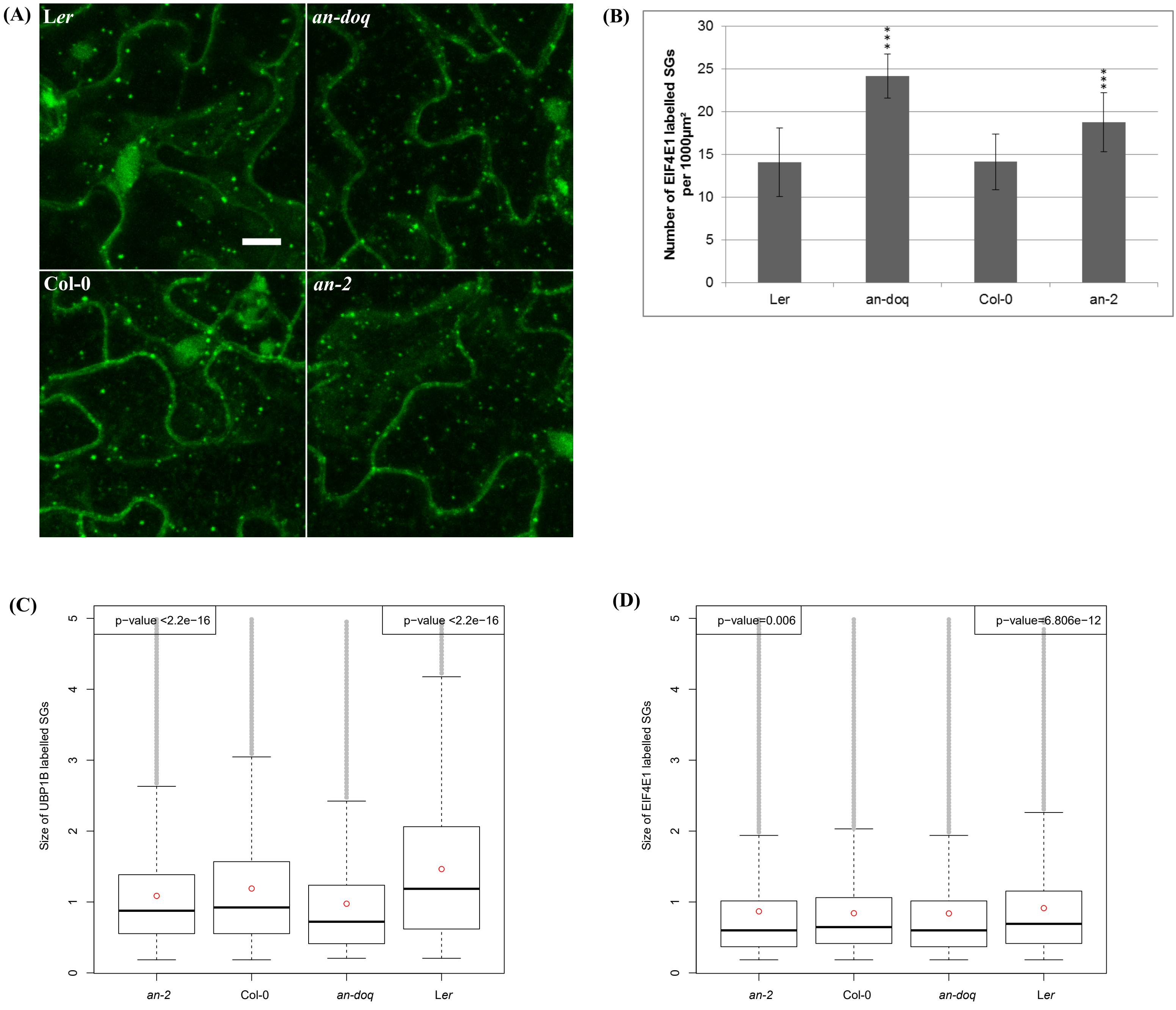

Figure S5: SG size and number is altered in *an* mutants

Transgenic lines expressing 35S:YFP-EIF4E1 or 35S:YFP-UBP1B in L*er*, *an-doq*, Col-0, *an-2* were analyzed after 40 minutes heat stress (39°C). The Z-stack images were analyzed for the number and size of SGs using ImageJ analyze particles tool. A) Confocal microscopy images showing formation of EIF4E1 labelled granules in L*er*, *an-doq*, Col-0 and *an-2.* Scale bar: 10µm. B) Number of EIF4E1 labelled SGs formed upon heat stress in wild type and *an* mutant backgrounds. Graphs show mean number ± SD per 1000μm^2 area for n=10~15 seedlings; * indicate p<0.001; student's t-test. C) Boxplots showing the size distribution (area in μm^2) of UBP1B labelled SGs formed upon heat stress in L*er*/Col-0 and *an-doq/an-2* mutant; student's t-test. D) Boxplots showing the size distribution (area in μm^2) of EIF4E1 labelled SGs formed upon heat stress in L*er*/ Col-0 and *an-doq/an-2* mutant; student's t-test. Straight lines within boxes represent median, red open circles represent mean value.**
