# Supplementary material for: ANGUSTIFOLIA, a Plant Homolog of CtBP/BARS Localizes to Stress Granules and Regulates Their Formation
Source: Front Plant Sci. 2017 Jun 13;8:1004. doi: 10.3389/fpls.2017.01004 (PMC5469197; doi:10.3389/fpls.2017.01004)
Supplement: Supplementary file 9 [file Image_6.pdf]

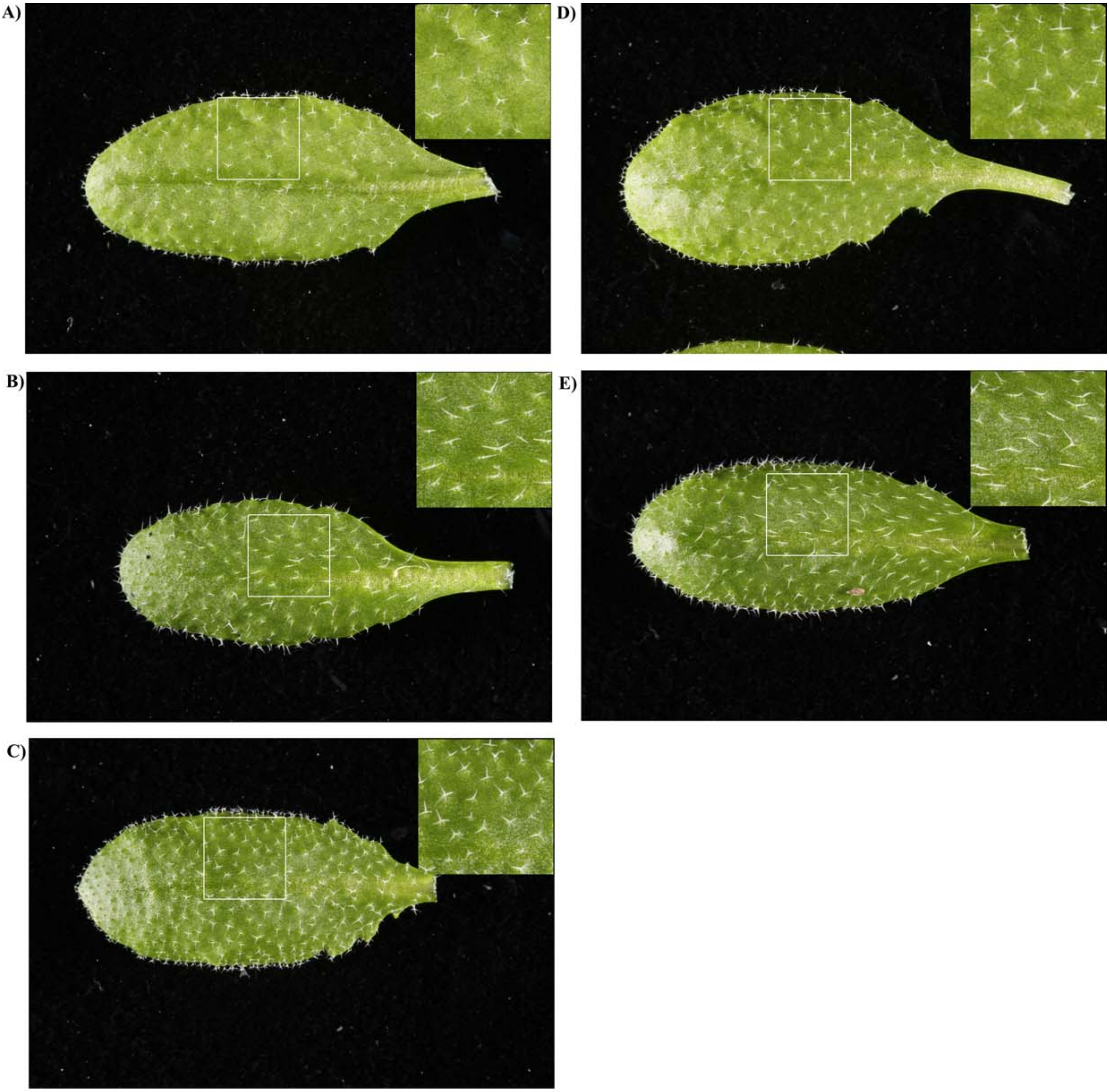

Figure S6: Phenotypes of lines expressing YFP-AN^{DOQ} and YFP-AN$^{GAD\rightarrow VVA}$ in *an-2* mutant background
A) Col-0 wild type B) *an-2* mutant C) *an-2 35S:YFP-AN* D) *an-2 35S:YFP-AN^{DOQ}* E) *an-2 35S:YFP-AN$^{GAD\rightarrow VVA}$*. For each line, at least 8 independent transformants were analyzed. The leaf length:width ratio was analyzed for a total of thirty leaves from 3 independent transformed lines. The ratio was 0.87±0.07 for Col-0, 1.6±0.14 for *an-2*, 1.04±0.07 for *an-2 35S:YFP-AN*, 0.99±1.2 for a*n-2 35S:YFP-AN^{DOQ}*, 1.23±0.24 for *an-2 35S:YFP-AN$^{GAD\rightarrow VVA}$*.
