# Supplementary material for: ANGUSTIFOLIA, a Plant Homolog of CtBP/BARS Localizes to Stress Granules and Regulates Their Formation
Source: Front Plant Sci. 2017 Jun 13;8:1004. doi: 10.3389/fpls.2017.01004 (PMC5469197; doi:10.3389/fpls.2017.01004)
Supplement: Supplementary file 10 [file Image_7.pdf]

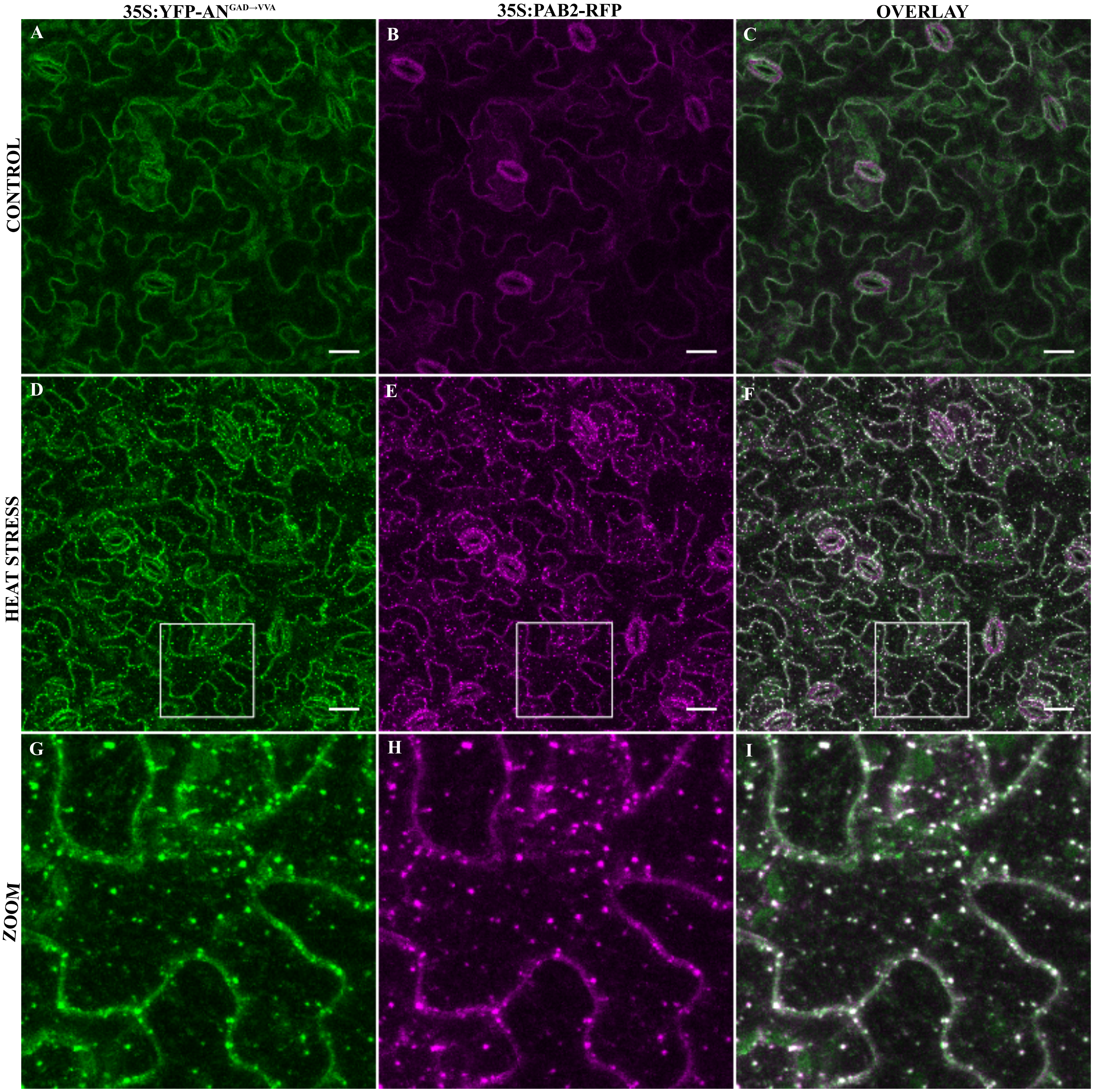

Figure S7: Co-localization of AN$^{GAD\rightarrow VVA}$ with PAB2

Transgenic lines expressing YFP-AN$^{GAD\rightarrow VVA}$ and PAB2-RFP. YFP- AN$^{GAD\rightarrow VVA}$ and PAB2-RFP expressing leaf without stress (A-C), after 40 minutes heat stress (39°C) (D-F) and a higher magnification (G-I) of the box indicated in (D-F). Scale bar: 20µm.
