# Supplementary material for: ANGUSTIFOLIA, a Plant Homolog of CtBP/BARS Localizes to Stress Granules and Regulates Their Formation
Source: Front Plant Sci. 2017 Jun 13;8:1004. doi: 10.3389/fpls.2017.01004 (PMC5469197; doi:10.3389/fpls.2017.01004)
Supplement: Supplementary file 11 [file Image_8.pdf]

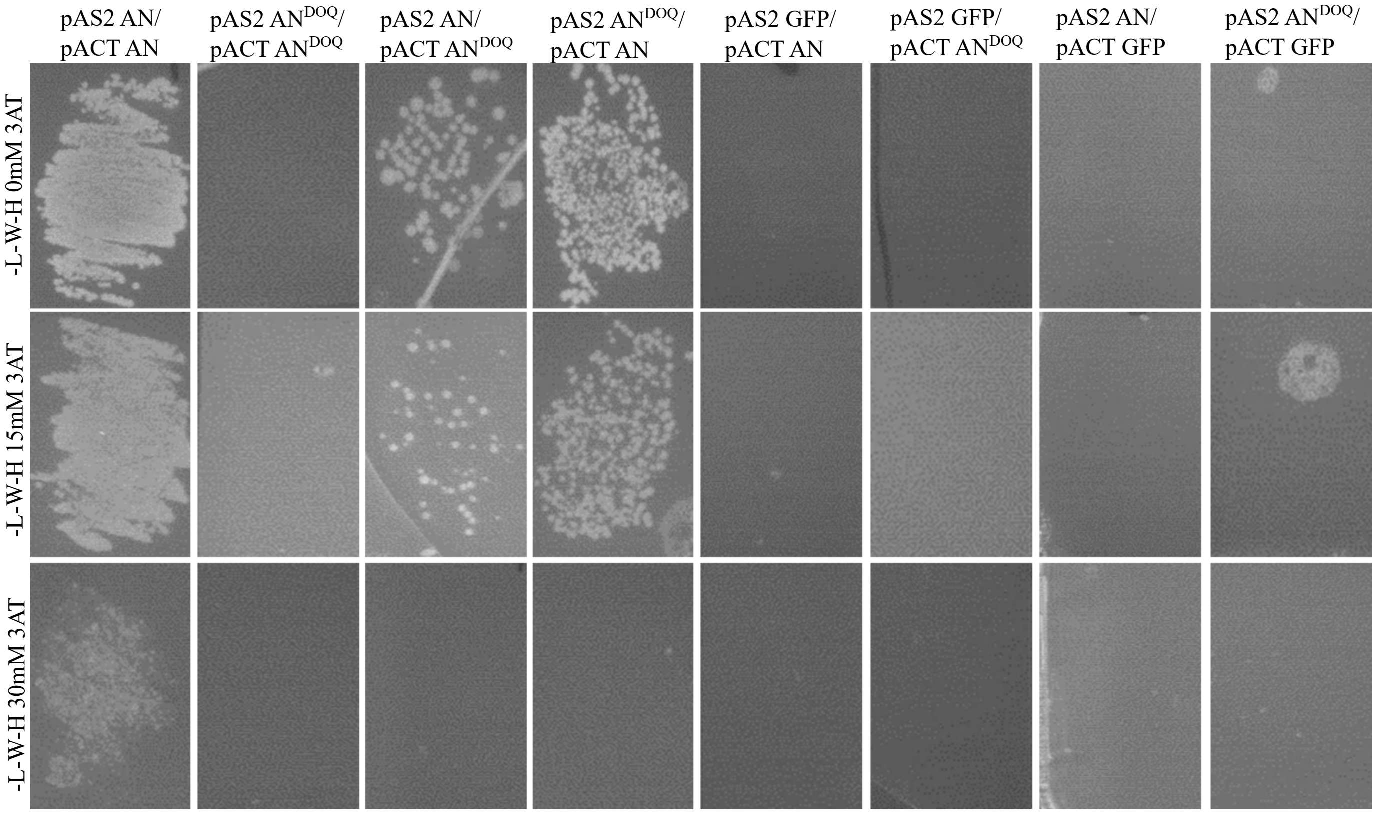

Figure S8: Comparison of dimerization efficiency of AN and AN^{DOQ} in yeast two-hybrid system Dimerization of AN-AN; AN^{DOQ}-AN^{DOQ}; AN-AN^{DOQ} was tested in a yeast two-hybrid assay at 0mM 3AT, 15mM 3AT and 30mM 3AT. GFP is the auto activation control.
